# Acute Stress Increases Sex Differences in Risk Seeking in the Balloon Analogue Risk Task

**DOI:** 10.1371/journal.pone.0006002

**Published:** 2009-07-01

**Authors:** Nichole R. Lighthall, Mara Mather, Marissa A. Gorlick

**Affiliations:** Davis School of Gerontology, University of Southern California, Los Angeles, California, United States of America; University of Parma, Italy

## Abstract

**Background:**

Decisions involving risk often must be made under stressful circumstances. Research on behavioral and brain differences in stress responses suggest that stress might have different effects on risk taking in males and females.

**Methodology/Principal Findings:**

In this study, participants played a computer game designed to measure risk taking (the Balloon Analogue Risk Task) fifteen minutes after completing a stress challenge or control task. Stress increased risk taking among men but decreased it among women.

**Conclusions/Significance:**

Acute stress amplifies sex differences in risk seeking; making women more risk avoidant and men more risk seeking. Evolutionary principles may explain these stress-induced sex differences in risk taking behavior.

## Introduction

Many of our decisions involve choosing whether to take a riskier action that has a larger potential reward or a safer, more conservative course of action. Sometimes, such decisions must be made under stress, such as stock trading decisions during a market crash or decisions about speeding through yellow traffic lights when late for a meeting. Recent studies have revealed that experiencing a stressor can change decision-making strategies and outcomes [1]–[5]. In particular, decisions that involve weighing risk versus reward may be affected by one's current stress level. Work inspired by Antonio Damasio's somatic marker theory [6] has demonstrated that bodily sensations signal the likely consequences of a risky action and help guide decision making [7]. The brain and the rest of the body are engaged in constant communication to maintain the body's dynamic equilibrium [8]. Stressful experiences threaten this homeostasis and elicit sympathetic nervous system responses and stimulate the release of cortisol [9]. These stress responses mobilize the body's resources to respond to a challenge while also activating feedback loops in the brain that help reinstate homeostasis. Of particular interest when considering stress effects on decision making, regions of the brain that play a key role in risk processing also are part of the core brain-body feedback loop [7] and are particularly responsive to stressful experiences [e.g.], [10,11]. Recent studies using neuroimaging show that acute stress influences activity within brain regions regulating homeostasis and emotions and that the activation in these regions correlate with circulating cortisol levels [12]–[14].

Biological sex is another factor that appears to influence risk taking. Greater risk taking in men than women has been observed across a wide range of behaviors. Compared with women, men make riskier investment decisions [15], [16], [17], have higher rates of alcohol abuse and dependence [18], and are more likely to die from violent deaths such as motor vehicle accidents [19]. These real-world differences in behavior may stem from sex differences in decision processing. In support of this proposition, d'Acremont and Van der Linden [20] compared risk-related decision making in adolescents and found that girls, but not boys, learned to make better decisions during the Iowa Gambling Task, in which selecting from risky decks of cards leads to greater overall losses [21]. Furthermore, some evidence indicates that when externally-provided risk taking goals are more difficult, males risk more than females, whereas the opposite is true when assigned risk taking goals are easy [22]. There are also sex differences in how much an individual's risk tolerance influences group decisions. Karakowsky and Elangovan [23] found that males are more risk tolerant and females more risk aversive in independent situations, but in mixed gender groups, males' risk tolerance more strongly influences the risk preferences of the larger group.

Thus, both stress and sex appear to independently impact risk taking tendencies. These two factors may also interact to influence risk-related decision making. Traditionally, the human stress response has been characterized as “fight-or-flight.” However, females show a different behavioral pattern in response to stress than males, one characterized as “tend-and-befriend” [24]. Using principles of natural selection, Taylor et al. argued that a stress response promoting aggressive behavior or fleeing may be adaptive for males but not for females given sex differences in parental roles. Females initially invest more in offspring through pregnancy, nursing and infant care, making females more vulnerable to external threats. Furthermore, if a mother attempted to attack a predator or flee in response to a threat, they would leave their offspring unprotected. Thus, it may be more adaptive for the stress response in females to inhibit risky responses such as fleeing or fighting a predator.

In the current study, we tested whether there are sex differences in how stress affects risk taking by having participants play the Balloon Analogue Risk Task (BART), a decision-making game which involves blowing up a simulated balloon on a computer screen [25]. Participants accumulate points each time they pump up the balloon, but each pump also carries the risk that the balloon will pop, leading the participant to lose all their points from that balloon. Performance on the BART is correlated with addictive, health and safety risk behaviors [26]–[28]. Participants in our study played the BART 15 minutes after experiencing either a stress challenge or a control task in order to synchronize the task with the stressor-related peak for the hypothalamic-pituitary-adrenal axis (HPA) hormone cortisol [29].

## Methods

### Participants

Forty-eight young adults (24 females) were recruited to participate in a study of stress and cognition and received either course credit or payment for participating. Three participants did not provide enough saliva for assay and were thus not included in subsequent analyses. The final sample included 23 females (11 stressed, 12 control; *M_age_* = 19.22, *SD* = 1.4) and 22 males (11 stressed, 11 control; *M_age_* = 21.95, *SD* = 4.2). No participants were using hormone birth control. In order to maintain stable cortisol levels, all participants avoided eating, smoking, exercising, and having caffeine within one hour of the study and avoided sleeping within two hours of the study. The study was approved by the University of California, Santa Cruz Institutional Review Board and written informed consent was obtained from all participants.

#### Computerized Risk Task

Participants' goal during the BART was to earn as many dollar points as possible [e.g., 25]. Participants were shown a mock list of high scores to provide a frame of reference for their performance, but no monetary reward was offered. During the BART, participants viewed a computer screen which displayed three items: a balloon with a button labeled *Click here to pump*, button labeled *Collect $$$*, and a box where total earnings were tallied in every trial. Every time the subject clicked on the “*pump*” button, the balloon increased slightly in size. When the *Collect $$$* button was pressed, the total earnings display added 5 cents for the current balloon. Each balloon in the 30 trials was set to explode at a random pump. If a balloon was pumped past its individual explosion point, a “pop” sound effect played and the participant did not earn any money for that balloon. At any point during a trial, participants could cash out by clicking the *Collect $$$* button and their earnings would be updated while a slot machine “payoff” sound emphasized the payment. The number of pumps before an explosion occurred ranged from 1–128. For every balloon, the first pump had 1/128 probability of exploding and a potential gain of 100% (i.e., from 5 cents to 10 cents), the second pump had a 1/127 probability of exploding and a potential gain of 50% (i.e., from 10 cents to 15 cents), and so on until the 128^th^ pump which carried a 1/1 probability of exploding and a potential gain of 0%. Thus, with each additional pump on a particular balloon the risk of losing increased and the relative gain decreased. In this way, some risk taking was necessary to make gains but excessive risk was associated with diminishing returns.

Participants did not receive information about the maximum number of pumps possible for balloons or the likelihood of explosions. Instead, participants were told: “It is your choice to determine how much to pump up the balloon, but be aware that at some point the balloon will explode. The explosion point varies across balloons, ranging from the first pump to enough pumps to make the balloon fill the entire computer screen.”

### Procedure

The study was conducted between 1400 and 1700 h to reduce the impact of circadian variability in cortisol levels. Participants were randomly assigned to the stress or control condition and were asked to drink an 8 oz bottle of water to ensure clean saliva samples. Ten minutes later a baseline saliva sample was collected. The cold pressor stress task was conducted by having participants submerge their non-dominant hand in a pitcher of ice water (0–3°C) for three minutes. The control task was conducted in the same manner using room-temperature water (22–25°C). Fifteen minutes after the cessation of the cold pressor task a post-stress saliva sample was collected, after which participants began the computerized risk task. Samples were immediately placed in labeled tubes and stored in a laboratory freezer at −30°C until they were shipped for assay.

## Results

To facilitate comparison among means, we report 95% confidence intervals; we also report partial eta squared (*η_p_^2^*) as a measure of effect size.

### Salivary Cortisol Response to Cold Pressor Stress

An ANOVA with sex and stress condition as between-subject factors revealed a main effect of stress group on cortisol change (15-min post-stress cortisol – baseline cortisol), *F*(1,41) = 6.05, *p*<.05, *η_p_^2^* = .13, such that stress participants' cortisol levels increased (*M_Δ_* = .14±.08 ug/dL) while control participants' cortisol levels did not change (*M_Δ_* = .00±.08 ug/dL). In addition, we observed an interaction between stress and sex for cortisol change, *F*(1,41) = 4.19, *p*<.05, *η_p_^2^* = .09, such that stress had a larger impact on cortisol change for women than for men (see Table 1). Examination of confidence intervals revealed that cortisol change was not reliably different for men in the stress and control groups, but women in the stress group had greater cortisol increases than women in the control group. At baseline there were no significant sex or stress group differences or interactions in cortisol levels.

**Table 1:** Cortisol change by sex and stress condition.

	No stress condition (warm water)	Stress condition (ice water)
**Sex**		
Male	.03±.11 ug/dL	.05±.11 ug/dL
Female	−.03±.10 ug/dL	.23±.11 ug/dL

Mean cortisol change (15-min post-water cortisol – baseline cortisol) after the ice or warm water hand immersion, with 95% confidence intervals.

### Balloon Analogue Risk Task (BART) Performance

For the BART, risk taking was measured by the average number of times a person pumped up a balloon before deciding to cash out [25]. This average was adjusted to exclude balloons that exploded, as the measure was curtailed for those balloons. An ANOVA with stress condition and sex as between-subject factors revealed no main effect of stress condition on the adjusted pump average, *F*(1,41)<.09, *p* = .76, *η_p_^2^* = .00, together with a main effect of sex as, overall, men took more risk than women, *F*(1,41) = 14.46, *p*<.001, *η_p_^2^* = .26 (*M_men_* = 45.46±3.63; *M_women_* = 35.89±3.55). However, this main effect was qualified by an interaction of stress condition and sex, *F*(1,41) = 5.94, *p*<.05, *η_p_^2^* = .13. Confidence intervals for this interaction indicated that risk taking was significantly higher among stressed males than among control males whereas risk taking was significantly lower among stressed females than among control females (see Table 2). Furthermore, men and women in the control group displayed similar levels of risk taking whereas in the stress group, men took significantly more risk than women.

**Table 2:** Risk taking by sex and stress condition.

	No stress condition (warm water)	Stress condition (ice water)
**Sex**		
Male	42.78±5.14	48.15±5.14
Female	39.34±4.92	32.45±5.14

Average number of pumps per balloon in trials without explosions (for all participants), with 95% confidence intervals.

To investigate the impact of cortisol on risk taking, cortisol change was included as a covariate in the risk taking analysis. The interaction between sex and stress condition for the number of balloon pumps did not reach significance after controlling for cortisol change, *F*(1,40) = 3.98, *p* = .05, *η_p_^2^* = .09. Cortisol responses to the cold pressor appear to account for a portion of the observed differences in risk taking between men and women in stressed and unstressed conditions; however, the effect size after cortisol was controlled for suggests that other factors were responsible for 9% of the variance in sex-dependant stress effects on risk taking. Correlations between cortisol change and adjusted pump average were then calculated for the whole group and for men and women separately. For the whole sample, there was a marginal inverse relationship between cortisol change and number of pumps, *r*(45) = −.29, *p* = .05, such that higher cortisol increases were related to more conservative behavior. This relationship between cortisol and pumps appears to be driven by females as cortisol change and number of pumps were negatively correlated for women, *r*(23) = −.43, *p*<.05, but not for men, *r*(22) = −.02, *p* = .92.

**Figure 1:**
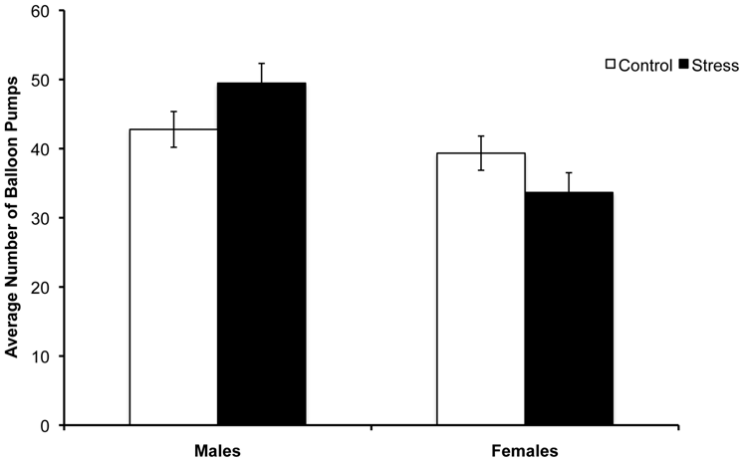
Interaction between sex and stress in risk taking. Average number of balloon pumps on trials without explosions for males and females who were equated for their cortisol stress response. Error bars represent standard errors.

As outlined above, women had a larger cortisol response to the cold pressor stress than men did. To test whether the sex by stress condition interaction for risk taking would hold up when cortisol responses in males and females were not significantly different, we removed the two males with the lowest cortisol change scores and the two females with the highest cortisol change scores among those in the stress condition, while keeping all the control participants. To confirm that the males and females in this group did not differ significantly in cortisol responses, we conducted an ANOVA examining cortisol change. As seen for the whole sample, there were significantly greater change scores in the stress condition (*M_Δ_* = .11±.07) than in the control condition (*M_Δ_* = .00±.06), *F*(1,37) = 5.12, *p*<.05, *η_p_^2^* = .12. Importantly, the interaction of stress condition and sex on cortisol change was no longer significant, *F*(1,37) = .89, *p*>.3, *η_p_^2^* = .02. Thus, among this subset of participants, the stress reactions for males and females were not statistically different. An ANOVA examining the average number of pumps on non-explosion trials for these participants revealed an interaction of stress condition and sex, *F*(1,37) = 5.26, *p*<.05, *η_p_^2^* = .12, which replicates the interaction seen among the broader group of participants. As shown in Figure 1, the sex difference in risk seeking was greater in the stress condition than in the control condition. This indicates that the sex differences in how stress affected decision making were not simply the result of sex differences in the intensity of the cortisol response to the stressor.

## Discussion

Many decisions involve choosing whether to risk something in the hopes of obtaining a potential reward or whether to take a safer course that reduces both the risk and opportunity for reward. In general, men tend to be more risk seeking than women [25], [30], [31]. For instance, in the United States, single women have a lower proportion of their wealth held as risky assets than do single men [16]. Our study suggests that acute stress amplifies sex differences in risk seeking, such that men become even more risk seeking and women more risk avoidant.

The results of the present study are in line with Taylor and colleagues' theory [24] that pressures of natural selection have resulted in different biobehavioral responses to stress in males (fight-or-flight) and females (tend-and-befriend). In pursuit of gains, men in our study took greater risk after stress – perhaps analogous to a “fight” response to stress exhibited in our male ancestors during competition for territory or other valuable resources. In contrast, women in our study were more conservative after stress – a beneficial response in early human females as risky pursuit of resources in mothers could endanger the lives of dependent offspring.

Although not examined here, it has been proposed that behavioral responses to stress are mediated by testosterone in males and by oxytocin in females. In males acute stress increases testosterone, and stress-related testosterone changes are predictive of aggression [32]. Aggression in females, however, does not appear to be enhanced by stress [see 24 for review]. Also, in contrast to typical fight-or-flight responses, oxytocin has been shown to exert calming effects [33]. This social hormone appears to be particularly important in determining the behavior of females as its effects are strongly modulated by estrogen [34], and oxytocin responses to stress are more commonly observed in females [e.g., 35]. In our study, cortisol reactivity to the cold pressor explained only 4% of the total variance in the sex by stress interaction on risk taking in the present study. Taken with the previous literature, this finding suggests that reproductive and social hormones may have determined the observed sex-specific effects of stress on risk taking to a greater degree than cortisol. Furthermore, the fact that we found a significant correlation between cortisol change and decision behavior in females but not males suggests that cortisol plays a larger role in how acute stress affects decision behaviors among females than among males.

An important point to note is that while taking more risk led to greater rewards in the Balloon Analogue Risk Task, risk-averse behavior may be beneficial in other decision scenarios. For instance, Preston and colleagues recently examined how social stress affects performance on the Iowa Gambling Task and found a non-significant trend in which stress made women select the decks with smaller risks and payouts (the optimal strategy) but exerted the opposite effect on men [4].

Future research should investigate how acute stress may modulate brain regions associated with decision making differently for males and females. Based on neuroimaging studies and data from patients with brain lesions, some of the key brain regions involved in decision making are the ventromedial prefrontal cortex, amygdala, anterior cingulate and insula. For instance, on laboratory tasks, patients with lesions in ventromedial prefrontal cortex [36], [37] and the amygdala [36], [38] fail to learn to avoid risky decks of cards that over time lead to greater losses than more conservative decks of cards. In addition, dopamine signaling in midbrain and striatal regions is thought to play a critical role in reward-related decision making [39]. To date, only one imaging study has examined neural activation during the BART [40]. In a version of the task adapted for functional magnetic resonance imaging (fMRI), reliable activation was found in the midbrain, anterior insula, striatum, dorsolateral prefrontal cortex, medial frontal cortex, and anterior cingulate during active risk taking.

Of particular relevance for our study, recent findings reveal that the effects of acute stress within brain regions implicated in risky decision making differ for males versus females. For instance, an fMRI study revealed that whereas acute psychological stress in men led to increases in cerebral blood flow in right prefrontal cortex and decreases in left inferior orbitofrontal cortex, acute stress in women primarily activated the ventral striatum, putamen, insula and cingulate cortex [41]. Likewise, a study examining fMRI activity during the anticipation and experience of visceral pain (a form of acute physiological stress) found that women showed greater activation in the amygdala, ventromedial prefrontal cortex and anterior cingulate, whereas men showed greater activation in the dorsolateral prefrontal cortex, insula and dorsal pons [42]. For women under stress, activity in the medial orbital frontal cortex and anterior cingulate were more positively correlated with amygdala activation than for males under stress [43]. Thus, acute stress seems to be more likely to activate the emotional and visceral network involved in decision making for women and more likely to activate dorsolateral and medial prefrontal regions in males. Because brain lesion studies have linked emotional and visceral processing structures with increasing risk avoidance and learning about task contingencies, greater activation in these regions may lead stressed women to respond more strongly to somatic cues to avoid risk than stressed men and help women learn more effectively about risk/reward contingencies. Conversely, males' greater prefrontal activation under stress may increase reliance on strategic processing rather than on somatic cues. Finally, as stress-induced drug cravings are associated with increased striatum activation [44] and sex differences in stress response also appear in striatal structures [41], this region may be a part of the neural mechanisms behind sex-dependent stress effects in risk taking.

This study raises several questions that are beyond the scope of the present findings. First, while cold pressor-induced changes to cortisol presented here are of a similar magnitude to those presented in other studies [e.g.], [45,46], we observed larger cortisol responses to cold stress in women versus men. The reason for this finding is unclear and the results are in contrast with several investigations showing that men are more likely to have enhanced HPA axis reactivity to psychological stress [see 47]. Furthermore, of the few studies investigating sex differences in cortisol reactivity to cold stress, some groups find greater cortisol responses in men [48] and others find no sex differences [45], [49]. Further research is warranted to determine whether there are reliable sex differences in HPA axis reactivity for some stressors and not others.

Another question for future research is whether psychological stress such as anticipating giving a speech would yield similar sex differences as the cold pressor stress manipulation that we used. Animal research has revealed two general stress pathways in the brain. One is a “systemic” pathway that elicits biological responses to stress by transmitting sensory information (threats to homeostasis such as pain) to regions directly innervating the paraventricular hypothalamic nucleus – a hub of the stress response system which initiates glucocorticoid release[see 50 for review]. Another “neurogenic” stress path is layered atop the simpler systemic pathway. Neurogenic stressors activate the paraventricular nucleus via forebrain structures in response to stimuli that are potentially threatening (e.g., predators, heights, social challenges); eventually resulting in a glucocorticoid response. Compared with stressors such as physical pain, psychosocial stressors thus may activate prefrontal and basal ganglia regions to a greater extent and may cause even greater interference with risk-related cognition. Forebrain structures, however, can also regulate paraventricular nucleus responses to systemic stressors in a top-down manner. For example, one study found that while anticipation of pain (psychological stress) activated emotion-arousal structures, delivery of pain resulted in activation of visceral afferent processing structures as well as cortical modulation of structures in frontal and parietal cortices [43]. Thus, systemic stressors may sometimes cause psychological stress and both types of stress involve higher cognition regions, but further research is needed to examine the extent to which their effects on the body and cognition are similar or different. One promising indication that these sex differences in the effects of stress on risky decision making generalize across stressors and decision contexts is that, like in our study, Preston et al. [4] found that social stress made females more conservative but males more risky on a gambling task (although their sex by stress interaction did not achieve statistical significance).

Cognitive neuroscientists are beginning to tease apart risk-related decision making into different categories with distinct neural correlates [e.g.], [40], [51,52]. Relative to other risk taking decision tasks, the decision properties and neural correlates of performance on the Balloon Analogue Risk Task have been understudied. For instance, whether decision making during the BART represents circumstances of risk (outcome is defined by a probability), ambiguity (outcome is not known at all), or some combination, is up for debate. Because the probability of the balloon popping is not known, the BART requires ambiguous decision making. However, whereas early trials of this task are clearly characteristic of ambiguous decision making (exploration), later trials may be more characteristic of risky decision making in which the probabilities are approximately known [53]. In any case, one valuable aspect of the BART is its predictive validity for real world behavior; the degree of risk seeking on the BART is correlated with risky behaviors such as gambling, smoking, unsafe sexual practices and illicit drug use [25], [28], [54], [55].

In closing, this study indicates that acute stress can enhance sex differences in risk taking behavior. Given that stress often accompanies decisions with risky alternatives, it is possible that stress contributes to sex differences in risk taking observed in society. Thus, an important avenue for future research on risky behavior is determining how social and biological factors may account for sex differences in risk taking under stress.
